# Mathematical modelling reveals potential acceleration of the supercontinent cycle

**DOI:** 10.1038/s41598-022-21662-x

**Published:** 2022-10-17

**Authors:** Arnaud Broussolle

**Affiliations:** 1 State Key Laboratory of Isotope Geochronology and Geochemistry, Guangzhou Institute of Geochemistry, Chinese Academy of Sciences, Guangzhou, 510640 China; 2 CAS Center for Excellence in Deep Earth Science, Guangzhou, 510640 China

**Keywords:** Geodynamics, Tectonics

## Abstract

The supercontinent cycle has been the focus of researchers for many years, but the parameters of its cyclicity remain a central debate; thus, prediction of the occurrence of the next supercontinent remains elusive. In this research, a mathematical point of view is adopted, based on the assumption that the supercontinent Columbia assembled at – 2000 Myr $\left( {X\left( { - 2} \right)} \right)$ and the supercontinent Rodinia assembled at – 1000 Myr $\left( {X\left( { - 1} \right)} \right)$. The younger supercontinents are calculated following this mathematical equation: $X\left( n \right) = 2*X\left( {n - 1} \right) - X\left( {n - 2} \right) - \left( {\frac{540}{{3^{n} }}} \right)$, where $X\left( n \right)$ represents the assembly and *n* is the position of the supercontinent in the sequence. Therefore, Gondwana $\left( {X\left( 0 \right)} \right)$ amalgamated at -540 Myr, Pangea $\left( {X\left( 1 \right)} \right)$ at – 260 Myr, Eurasia $\left( {X\left( 2 \right)} \right)$ at – 40 Myr and Pangea Proxima $\left( {X\left( 3 \right)} \right)$ might form at + 160 Myr. Moreover, two logarithmic regressions give fairly similar results, confirming that a constant acceleration of the supercontinent cycle is probable. The detrital zircon, metamorphic and hafnium isotope records support the assemblies’ hypotheses that produce the mathematical equation. However, a recent supercontinent or “megacontinent” called Eurasia lacks strong geological evidence in the three datasets. These findings might reconcile the paradox brought about by the closer ages in time for the Earth’s more recent supercontinental assemblies and the assumed constant cyclicity of the cycle.

## Introduction

**Figure 1:**
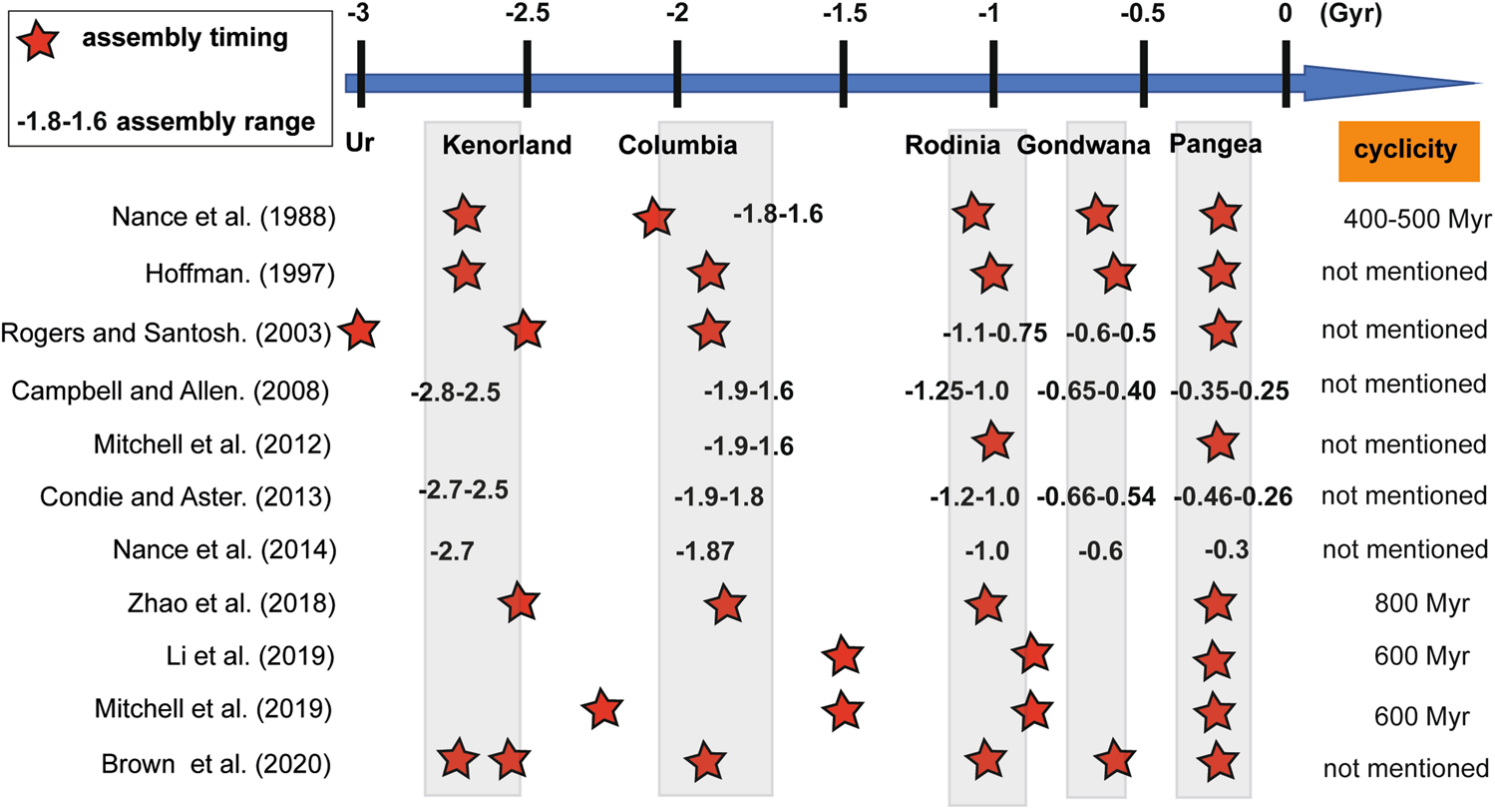
Chronological scale showing the different supposed supercontinent assemblies based on the literature (red stars denote the timing of assemblies whereas ages show ranges of assemblies). Based on the literature, 4 supercontinents can be recovered named Columbia, Rodinia, Gondwana and Pangea, whereas Ur and Kenorland represent possible Archean supercontinents or supercratons. Gyr refers to giga years whereas Myr refers to million years. The cyclicity represents a duration in Myr. The references are listed in the research article.

The supercontinent cycle (termed Sc) is one of the strongest paradigms in plate tectonics proposed by Wegener^1^ in 1912 and has since been corroborated by many studies^2–5^. The cycle encompasses collision followed by breakup-oceanic spreading-oceanic subduction and collision forming a comprehensive theory for Earth evolution i.e.,^2^. To date, its cyclicity (collision to collision) is assumed to be approximately 400–600 Myr in duration (Fig. 1)^2,3,5–7^. However, several recent authors proposed a cyclicity of approximately 800 Myr, suggesting that Gondwana was not a complete supercontinent^4,8,9^. This variety of assumed ages shows that the supercontinent cycle is not fully understood and that its frequency and periodicity remain debatable questions in modern geology^10^. For example, the latest supercontinent Pangea was formed at – 250 Myr, creating a single landmass that broke up starting at approximately – 180 Myr, separating major continents and opening major oceans worldwide besides the Pacific Ocean^2,11^. It is thus presumed that Pangea Proxima, the next supercontinent will be assembled at 200–250 Myr in the far future^2,6,11,12^. There is a strong likelihood that the succession of each previous supercontinent assembly could be used to recover the pattern of supercontinent cyclicity. Consequently, it is speculated that a mathematical equation prediction may fit the observations in Earth’s supercontinent history. Nonetheless, Earth has evolved since − 4550 Myr and it is consequently very difficult to establish the exact timing of Earth’s previous supercontinent assemblies because of several destructive cycles (such as possibly forming Kenorland, Columbia, Rodinia, Gondwana and Pangea) (Fig. 1)^7,13–18^. To fulfil this end, the Earth’s metamorphic record is used as a proxy for supercontinental assemblies, considering that metamorphic events result mostly from collision and the closure of oceanic domains (Figs. 1, 3)^18–20^. In addition, the detrital zircon record is also used as a verifying proxy for supercontinental assemblies, which shows that increased sediment supplies may have occurred at each assembly (Figs. 1, 3)^17^. Indeed, the formation of supercontinents leads to increased mountain belts, high topography and subsequent high erosional rates, e.g.,^21^. Finally, the ε_Hf_(t) record could be considered a validating proxy for supercontinental assemblies because increased crustal reworking could occur during the assembly of a supercontinent (Fig. 3), e.g.,^22^. These three potential proxies allow the establishment of the approximate assembly dates of the different supercontinents, at − 2700 to − 2500 Myr for Kenorland, − 2100 to − 1600 Myr for Columbia, at − 1300 to − 900 Myr for Rodinia, at − 660 to − 450 Myr for Gondwana, and at − 350 to − 230 Myr for Pangea (Figs. 1, 3)^4,9,10,13–20,22–25^. Given all these elements and based on the assumption of the assembly of Columbia and Rodinia, a mathematical equation can predict the youngest supercontinental assemblies and suggest a date for the assembly of the future Pangea Proxima. The corresponding equation and its results are introduced in the next section and explained in the Methods section.

## Mathematical equation results and error assessment

In this section, the previous supercontinental assemblies are used to recover the different supercontinental cycles $\left( {Sc\left( n \right)\;{\text{for}} \;n = - 1;0;1;2;3} \right)$. Considering the dates of assembly for Columbia $(X\left( { - 2} \right)$ and Rodinia $(X\left( { - 1} \right)$, an assembly date of Gondwana is proposed $\left( {X\left( 0 \right)} \right)$, which is subsequently used to predict the youngest supercontinents $\left( {X\left( n \right)\;{\text{for}}\;n = 1;2;3} \right)$ (i.e., Pangea, Eurasia and in the future Pangea Proxima).

The calculation process of the date of a given supercontinent assembly $X\left( n \right)$ depends on the following equation (all methodological and mathematical details regarding Eqs. (4) and (5) are found in the Methods section):

1 $$X\left( n \right) = 2*X\left( {n - 1} \right) - X\left( {n - 2} \right) - \left( {\frac{540}{{3^{n} }}} \right)$$

where $X$ represents the supercontinent assembly in Myr. The value *n* represents the position of the supercontinent in the sequence of assemblies. The constant $540\;Myr$ represents the first cycle acceleration $A\left( 0 \right)$ and 3 the acceleration constant of the cycle. The initial assemblies are Columbia formed at $X\left( { - 2} \right) = - 2000$ and Rodinia formed at $X\left( { - 1} \right) = - 1000.$ From Eq. (1) the younger supercontinents are calculated and Gondwana formed at $X\left( 0 \right) = - 540;$ Pangea at $X\left( 1 \right) = - 260$ and Eurasia at $X\left( 2 \right) = - 40$; thus Pangea Proxima $\left( {X\left( 3 \right)} \right)$ could be assembled at + 160 Myr. The past and future durations of the cycles (Sc) are found with Eq. (4) and hypothetical future assemblies $X\left( 3 \right)\;{\text{to}}\;X\left( 7 \right)$ are also recovered with Eq. (1) (see Table 1 for results).

**Table 1:** Assembly dates (*X(n*)) and corresponding duration of supercontinent cycles (*Sc*(*n*)) (in Myr).

X(− 1)	− 1000	Sc(− 1)	1000
X(0)	− 540	Sc(0)	460
X(1)	− 260	Sc(1)	280
X(2)	− 40	Sc(2)	220
X(3)	160	Sc(3)	200
X(4)	353.3	Sc(4)	193.3
X(5)	544.4	Sc(5)	191.1
X(6)	734.8	Sc(6)	190.3
X(7)	924.9	Sc(7)	190.1

Different values of A(0) and the constant of acceleration were investigated to test the robustness of the initial parameters. The results show that the initial acceleration can be in the range of 540 $\pm$ 10 Myr without propagating to many errors on the predictions (Table 2). These results show that each iteration *n* increases the range of the ages for the assemblies, such that the prediction for *n* = 4 has 135.2 Myr spreading. Although the predictions are widespread in ages, they still provide a good estimate for each assembly. Thus, the ages for A(0) in Table 2 have to be taken has upper and lower limits of the predictions if the first acceleration A(0) and thus the first assembly ranges between − 530 to − 550 Myr. It is noteworthy that the first acceleration of 540 Myr provides the best predictions for the assemblies of supercontinents. The acceleration constant of 3 was investigated between 2.95 and 3.05 and the results are portrayed in Table 2. The results show that the first prediction is reasonable, but the subsequent calculations for *n* = 2;3;4 are too widespread. Thus, for *n* = 4, $X\left( 4 \right)$ ranges between 226 and 611 Myr. These results are not acceptable because of overlapping $X\left( 3 \right)$ and $X\left( 5 \right)$ assemblies. These observations show that the constant of acceleration cannot fluctuate in the mathematical equation and that the constant 3 provides the best calculations for the predictions of the supercontinental assemblies.

**Table 2:** Investigation on the initial parameters of mathematical Eq. (1).

Parameters	Constant A(0)		Constant of acceleration	
	550	530	2.95	3.05
X(1)	− 283.3	− 236.7	− 263.1	− 253.8
X(2)	− 77.7	− 2.1	− 107.9	43.2
X(3)	107.5	212.8	56.2	287.3
X(4)	285.9	421.1	226.0	611.7

**Figure 2:**
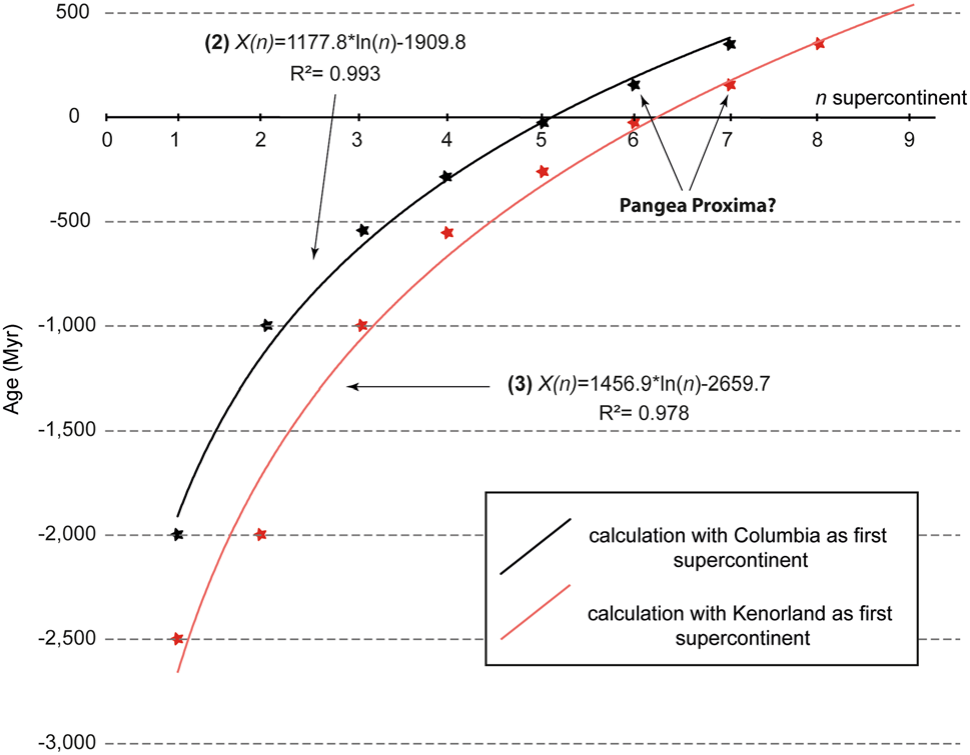
Plot of age of assembly versus *n* supercontinent cycles. It shows that the mathematical Eq. (1) results for *n* = (1;2;3;4;5;6;7;8) are consistent with two logarithmic regressions with a fairly good correlation factor. Positive ages represent dates in the future. The assembly results of the two regressions are portrayed in Table 3.

To assess the assembly error of mathematical Eq. (1), the assembly results are plotted on an age diagram versus *n* consecutive supercontinent cycles (Fig. 2). The plot demonstrates that the calculated assemblies with Eq. (1) fit two logarithmic regressions, whether two different first supercontinents are considered for Earth’s geodynamic evolution. The two regressions and their parameters are calibrated by using the assembly results of Eq. (1) which are provided in Tables 1 and 3. Equation (2) only considers the results from Eq. (1), suggesting that Columbia could be the first supercontinent in Earth history (Table. 3; Fig. 2), where $X\left( n \right)$ is still the supercontinent assembly and *n* is the position of the supercontinent in the sequence:

2 $$X\left( n \right) = 1177.8*\ln \left( n \right) - 1909.8\;R^{2} = 0.993$$

**Table 3:** Error assessment for mathematical equations on the prediction of supercontinent assembly (in Myr).

Supercontinent	Estimated assemblies based on the three proxies	Equation (1) results	Equation (2): X(n) = 1177.8 * ln(n)− 1909.8	Equation (1) results with Kenorland	Equation (3): X(n) = 1456 .9 * ln(n)− 2659.7
Kenorland	− 2700 to − 2500	–	–	− 2500	− 2660
Columbia	− 2100 to − 1600	− 2000	− 1910	− 2000	− 1649
Rodinia	− 1300 to − 900	− 1000	− 1093	− 1000	− 1059
Gondwana	− 660 to − 450	− 540	− 615	− 540	− 640
Pangea	− 350 to − 230	− 260	− 277	− 260	− 314
Eurasia	–	− 40	− 14	− 40	− 49
Pangea Proxima	200–250	160	199	160	175
+ 1	–	353.3	381	353.3	369

Equation (3) considers the results from Eq. (1) with the addition of a supercontinent Kenorland at approximately − 2500 Myr, which could be argued as the potential first supercontinent (Table. 3; Fig. 2)^4,5,14,26^, where $X\left( n \right)$ is still a supercontinent assembly and *n* is the position of the supercontinent in the sequence:

3 $$X\left( n \right) = 1456.9*\ln \left( n \right) - 2659.7\;R^{2} = 0.978$$

The predictions of the two logarithmic regressions are portrayed in Table 3 and show that the results are fairly consistent with the predictions of mathematical Eq. (1). It predicts Columbia, Rodinia, Gondwana, Pangea, Eurasia and Pangea Proxima assemblies at − 1910 to − 1650 Myr; − 1093 to − 1060 Myr; − 615 to − 640 Myr; − 277 to − 314 Myr; − 14 to − 50 Myr and 199 to 175 Myr, respectively (Table. 3). Furthermore, the correlation coefficient R^2^ (R^2^(2) = 0.993 and R^2^(3) = 0.978) shows a good fit for the calculations of mathematical Eq. (1). When considering Columbia as the first supercontinent, the assembly occurs at − 1910 Myr. However, when considering Kenorland as the first supercontinent, it implies that the assembly of Columbia is delayed until − 1650 Myr, quite far from the hypothesized value of − 2000 Myr. The assemblies of Rodinia, Gondwana, and Pangea are fairly consistent with the results from Eq. (1) showing slightly earlier assembly ages for the three of them (Table. 3). The assembly of what is called Eurasia (referring to a “megacontinent” from Wang, et al.^12^) is between − 50 and − 14 Myr. These ages range in the formation of the Alps and the Himalaya orogenic belts in the Cenozoic. To date, it is quite puzzling why our recovered cyclicity falls in the age of these two collisional belts, and their relationships within the supercontinent cycle are questionable. Finally, the two regressions predict Pangea Proxima assembly between 175 and 200 Myr, which is slightly earlier than the literature assumptions between 200 and 250 Myr e.g.,^2,6,11^. Thus, the discrepancies between the predictions and the literature are explained by the possible acceleration of the supercontinent cycle. It is thus specified that the name Pangea Proxima does not refer to the mode of gathering of the next supercontinent^11^ but factually that the next supercontinent gathering could occur earlier than the literature assumptions.

As mentioned in the introduction, the supercontinent cycle is considered between assembly and assembly, irrespective of which oceans closed or which blocks amalgamated. Indeed, it is accepted that Wilson cycles and supercontinent cycles can be set as in or out of phases e.g.,^6,11^. Thus, conceptually, the calculated supercontinent cyclicity is recovered from the collision to the collision of each supercontinent. According to the supposed collisions (− 2000 and − 1000 Myr) and the recovered assemblies (− 540, − 260 and − 40 Myr), it is reasonably presumed that the supercontinent cycle is accelerating. Consequently, the calculation contrasts with the usual steady durations of the supercontinent cycle of 400 to 800 Myr (Fig. 1), e.g.,^2–6,8,11^ and favours a speeding rate. This perspective helps reconcile the closer ages for the supposed Earth’s supercontinent assemblies (− 2100 to − 1600 Myr for Columbia, at − 1300 to − 900 Myr for Rodinia, at − 660 to − 450 Myr for Gondwana and at − 350 to − 230 Myr for Pangea (Fig. 1)). In this paper, a mathematical equation was created to fit the observations in Earth’s supercontinent history. This suggests that the cycle could accelerate at each assembly (Eqs. (4) and (6), see the Methods section for details). It therefore allows the calculation of future assemblies, probably reaching a critical supercontinent cyclicity of 190 Myr (after $n = 7; {\text{i}}.{\text{e}}.,{\text{ A}}\left( 7 \right) = \left( {\frac{540}{{3^{7} }}} \right) = 0.25{\text{ and Sc}}\left( 7 \right) = 190.1)$. In addition, the equation potentially predicts the next supercontinent assembly at + 160 Myr for Pangea Proxima, which is at least 40–90 Myr earlier, than the usually assumed assembly of Pangea Proxima.

## Discussion

### Correlation between mathematical results and geological proxies

**Figure 3:**
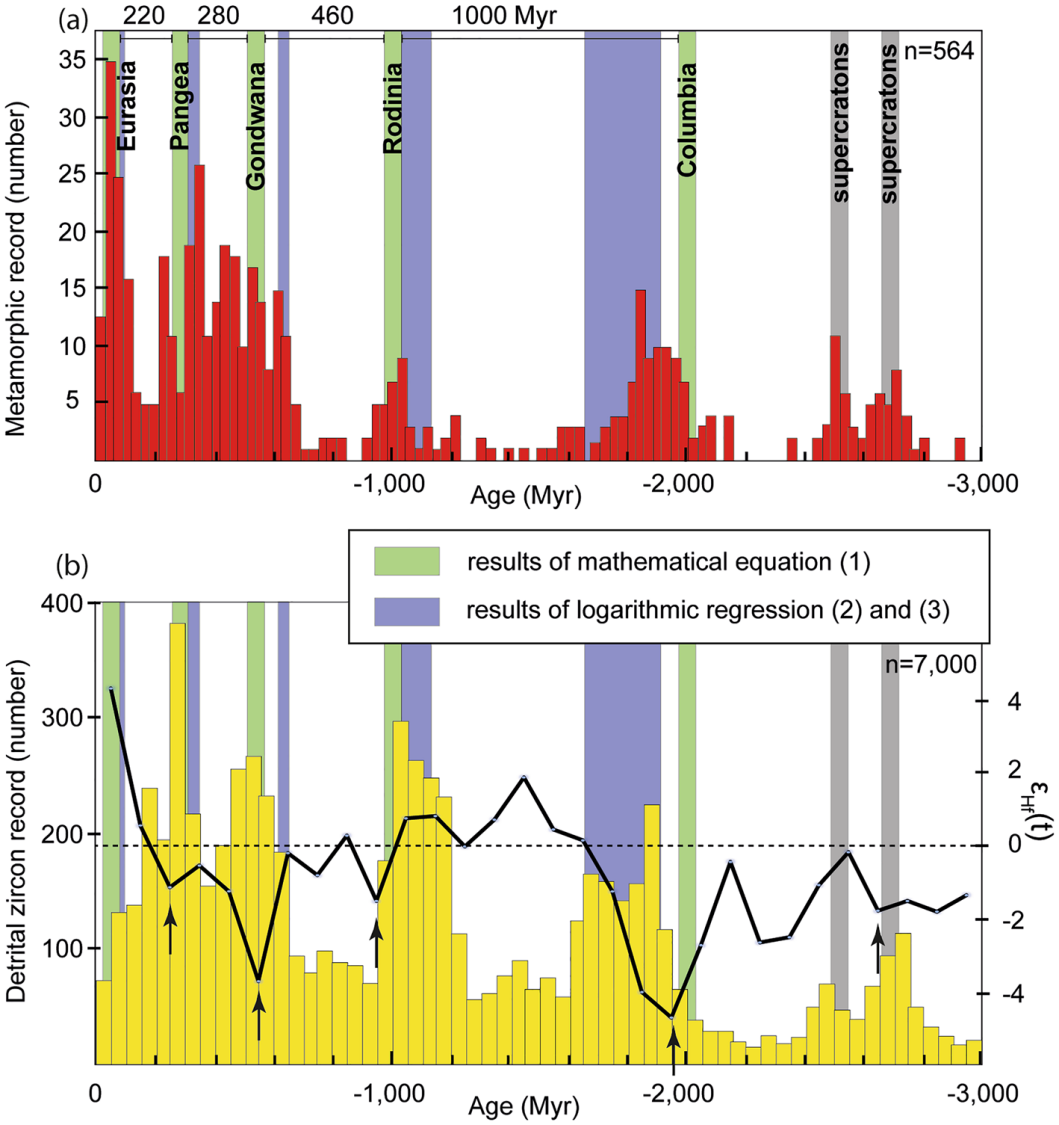
**a** Probability density plot for the Earth’s metamorphic record adapted from Brown, et al.^19^ (database in Brown and Johnson^18^). **b** Probability density plot for the detrital zircon record adapted from Campbell and Allen^17^ superposed with the ε_Hf_(t) record taken from Puetz and Condie^29^. The ε_Hf_(t) record (black curve, with right hand side axis) is plotted as the mean value for each 100 Myr bin. The different inferred assemblies obtained in this study are portrayed in **a** and **b** and show relatively good matches with the three geological proxies.

To test the age of assembly of each supercontinent and the plausibility of the acceleration of the supercontinent cycle, the proxies of the metamorphic, detrital zircon and ε_Hf_(t) records are plotted on a diagram from − 3000 Myr to present (Fig. 3a,b). The metamorphism database is a consistent proxy for the assembly of supercontinents e.g.,^19,20^. It shows important record at − 2700 to − 2500, at − 2100 to − 1700, at − 1200 to − 900, at − 700 to − 200 and at − 100 to − 20 Myr (Fig. 3a). The detrital zircon database shows increased sediment record at − 2800 to − 2400, at − 1900 to − 1600, at − 1250 to − 1000, at − 650 to − 450 and at − 350 to − 250 Myr (Fig. 3b). Interestingly, there is no increased sedimentary records from − 100 Myr to the present. The ε_Hf_(t) dataset varies from approximately − 5 to + 5 and show negative peaks at − 2650, − 1950, − 950, − 550 and − 250 Myr, which are considered acceptable proxies of supercontinental assemblies, because increased crustal reworking could occur during supercontinent gathering e.g.,^12,13,22,27^. However, the ε_Hf_(t) dataset from − 200 Myr to present is positive, suggesting that crustal growth predominated against crustal reworking.

Discussing the three geological proxies independently would not help to assess the results of the mathematical equation, as it shows different periods for the assembly of each supercontinent, although some proxies overlap in time (especially for Columbia). However, when combined, it shows that the assembly of Kenorland is not truly supported by the three databases. The two detrital zircon peaks at − 2700 and − 2500 Myr obtained for the Neoarchean match two separated supercratons (Fig. 3a,b). The metamorphic record formed at − 2700 to − 2500 Myr shows two peaks, which could also be consistent with two separate supercratons. In addition, the ε_Hf_(t) record is not distinctive from − 3000 to − 2500 Myr. These observations suggest that the assumption of Columbia as the first supercontinent in Earth history in this research is reasonable. However, it is not possible to conclude whether Kenorland or Columbia is the first supercontinent in Earth history because the two logarithmic regressions give fairly good correlation factors (Fig. 2).

The three geological records show that the Columbia and Rodinia assemblies could have occurred at − 2100 to − 1600 Myr and at − 1300 to − 900 Myr (Fig. 3a,b). Nevertheless, the ε_Hf_(t) record peaks negatively at approximately − 1950 and − 950 Myr, which are good matches with the initial hypothesis for the assembly of the supercontinent at − 2000 and − 1000 Myr. In addition, the ε_Hf_(t) records show two negative peaks at approximately − 550 Myr and at − 250 Myr, thus supporting the assembly of Gondwana and Pangea (Fig. 3b). Even if many researchers argue that Gondwana is formed of two landmasses, called East and West Gondwana^4,6,8,12^, which only joined during the assembly of Pangea, the equation presented here suggests that Gondwana could be considered a supercontinent similar to the other landmasses^10,13,14,19,24^. Thus, it is considered that the supposed and recovered assemblies $X\left( { - 2; - 1;0;1} \right) = \left( { - 2000; - 1000; - 540; - 260 } \right)$ are acceptable estimates for the assembly of the respective supercontinents. To conclude, if the equation predictions and logarithmic regressions are coherent, they could provide an average age for the formation of each supercontinent, thus providing a robust prediction for the assembly of the next supercontinent Pangea Proxima. Thus, it is suggested that beginning with the assembly of Columbia, the supercontinental cycle was accelerating. The mathematical Eq. (1) could reconcile the cyclicity with closer ages in time for the Earth’s more recent supercontinental assemblies. The acceleration of the supercontinent cycle does not necessarily imply that the plate tectonic speed is getting faster over time. Indeed, there is no consensus towards the speed of plate tectonics in the past and it has been argued that plate velocities could have been faster but also slower in the past^9,28^.

### Implications for the supercontinent cycle

The case for Gondwana (or Pannotia) considered a complete supercontinent is controversial e.g.,^4,6,8,10,16,20^. If its existence is supported, as assumed in this paper, it would have a profound influence on the mantle geodynamics and the supercontinent cycle. The three geological databases, show that Gondwana has strong geological evidence between − 600 and − 450 Myr. Indeed, the metamorphic record is increased from − 700 to − 400 Myr, the sedimentary record peaks from − 650 to − 450 Myr, and the ε_Hf_(t) record shows a negative peak at − 550 Myr (Fig. 3a,b). These increased geological records are collectively taken as resulting from the assembly of a supercontinent in the Ediacaran to Fortunian. Murphy, et al.^10^ also considered the assembly of Gondwana in that period of time, which is also emphasized by the results in this study (− 640 to − 540 Myr), e.g.,^21^. However, Wang, et al.^12^ considered the important negative ε_Hf_(t) peak in the Cambrian as an indicator for the formation of a “megacontinent” called Gondwana preceding the assembly of Pangea. Their findings are also supported by the assembly of the megacontinent Nuna before the assembly of the supercontinent Columbia (e.g., negative ε_Hf_(t) peak at − 1,950 Myr). However, in this study, the negative ε_Hf_(t) peaks are considered assemblies of supercontinents (i.e., − 1,950, − 950, − 550 and − 250 Myr) and fit the two other geological proxies (Fig. 3a,b). A mathematical investigation of coupling between megacontinent preceding the assembly of supercontinent was performed using the same logarithmic regression methodology as in this study (supplementary material S1) and it revealed that an acceleration of the supercontinent cycle is also observed with the megacontinent hypothesis. However, the acceleration is slower and not constant in comparison with the acceleration of 3 recovered from Eq. (1). More importantly, it also predicts Pangea Proxima at approximately 160 Myr (see supplementary material S1). Consequently, this study supports the assembly of Gondwana, which probably had profound influences on sedimentary, metamorphic and magmatic records, as envisaged by many authors (Fig. 3a,b), e.g.,^17,21–24^. Therefore, omitting the Gondwana supercontinent, could imply missing one cycle for the supercontinent evolution. These aspects might have profound implications for the supercontinent cycle and geodynamic models of Earth, e.g.,^10^.

The acceleration of the supercontinent cycle hypothesis implies an assembly of a supercontinent at approximately − 40 Myr called Eurasia. This assembly is also supported by the logarithmic regressions at − 50 to − 14 Myr (Table. 3 and Fig. 2). Supercontinents are characterized by the assembly of the majority of landmasses (70 to 80%), with a possible tenure of at least 100 Myr, i.e.,^10^. If this is mostly valid for Earth’s previous supercontinents (e.g., Columbia, Rodinia, Gondwana and Pangea), the case of Eurasia is puzzling. Although several cratonic terranes were accreted during the closure of the Neo-Tethys Ocean (i.e., Arabia and India), major terranes are still separated by the Atlantic, Pacific and Indian Oceans (i.e., Africa, Antarctica, Australia, North America and South America). Therefore, the landmass of Eurasia only covers 36% of Earth’s surface, which precludes it from being a supercontinent. In addition, its duration is difficult to quantify because the assembly might have occurred only at − 40 Myr.

The metamorphic record peaks after − 50 Myr, the detrital zircon record is almost absent in the last 100 Myr and the ε_Hf_(t) record shows a juvenile signature during the last 200 Myr (Fig. 3a,b). The closure of the Neo-Tethys Ocean occurred in the Cenozoic and is observed in the metamorphic record. However, the two other geological proxies mostly show contrasting evolution with the other supercontinents. Based on all these data, the assembly of a supercontinent in the Cenozoic is questionable. The assembly of Eurasia closes the Neo-Tethys Ocean terminating its Wilson cycle and could match a supercontinent cycle. However, it falls between the Wilson cycle of the Atlantic or the Pacific Oceans. Indeed, closing the Atlantic Ocean at approximately 160–200 Myr would result in a Wilson cycle duration of 340–380 Myr, consistent with a recent study^5^. However, considering the results in this study, there could actually be two supercontinent cycles (i.e., Sc (2) = 220 and Sc(3) = 200 Myr, Table 1) within the complete Wilson cycle of the Atlantic Ocean. Thus, if these two cycles are grouped, a supercontinent cycle duration of approximately 420 Myr would occur between Pangea and Pangea Proxima, which is almost consistent with the initial theory of supercontinents (400–500 Myr cyclicity, Fig. 1)^2,3^. In summary, supercontinental cycles and Wilson cycles may coincide or may not coincide during Earth evolution. Therefore, studies on the modes of formation of the supercontinent and Wilson cycles will be critical to better understand the Earth’s geodynamic evolution but require knowing precisely the numbers of previous supercontinents.

## Conclusions

- The mathematical equation and the two regressions obtained in this study give suitable predictions for the assemblies of Gondwana, Pangea and Pangea Proxima and suggest that the supercontinent cycle might accelerate.
- This paper suggests that the assembly of Pangea Proxima will occur slightly earlier than common predictions at approximately + 160 to + 200 Myr. These results could be used to test the different competing models of assembly for the next supercontinent gathering.
- This study cannot conclude whether the oldest supercontinent is Kenorland or Columbia although considering Columbia gives a more robust logarithmic regression.
- The predicted supercontinent called Eurasia (− 40 Myr) is questionable and lacks strong geological evidence on the three geological proxies.

## Methods

The implementation of the mathematical equation is performed by following this methodology. First, it is considered that the cyclicity of the supercontinent accelerates, therefore reducing the duration of the cycle between two complete assemblies. An acceleration parameter A is consequently added to the mathematical equation of the theoretical cyclicity.

4 $$X\left( n \right) = X\left( {n - 1} \right) + Sc\left( {n - 1} \right) - A\, with\, n \in {\mathbb{Z}} \left\{ {\begin{array}{*{20}c} { + \infty } \\ 0 \\ \end{array} } \right.$$

where X is an assembly, Sc is a supercontinent cycle and A is the probable acceleration of the supercontinent cycle. Then, it is assumed that a supercontinent cycle (Sc), corresponds to the duration between two consecutive assemblages of supercontinents. This assumption allows us to write $Sc\left( n \right)$ as:

5 $$Sc\left( n \right) = X\left( n \right) - X\left( {n - 1} \right)$$

To perform a substitution in Eq. (4), it is observed that $Sc\left( {n - 1} \right) = X\left( {n - 1} \right) - X\left( {n - 2} \right)$; therefore, Eq. (4) becomes Eq. (6):

6 $$X\left( n \right) = 2*X\left( {n - 1} \right) - X\left( {n - 2} \right) - A$$

In the second part of the investigation, several ages of the oldest supercontinent’s assemblies are studied. Thus, it is observed that $X\left( { - 2} \right) = - 2000$ and $X\left( { - 1} \right) = - 1000$, produce Eq. (6) with this solution for $X\left( 0 \right)$: $X\left( 0 \right) = 2*X\left( { - 1} \right) - X\left( { - 2} \right) - A\left( 0 \right) = - A\left( 0 \right)$.

Thus, $$X\left( 1 \right) = 2*X\left( 0 \right) - X\left( { - 1} \right) - A\left( 1 \right) = 2* - A\left( 0 \right) - \left( { - 1000} \right) - A\left( 1 \right) = - 2*A\left( 0 \right) + 1000 - A\left( 1 \right)$$

Then, different ages for $X\left( 0 \right)$ and $X\left( 1 \right)$ are examined, so that the assembly occurs in the ranges of the literature for Gondwana (i.e., between − 660 and − 500 Myr) and Pangea (i.e., between − 350 and − 230 Myr)^10,14,16,21^. This part of the process is performed empirically, so that $X\left( 0 \right)$ and $X\left( 1 \right)$ produce the best match for the assemblies from the literature for both Gondwana and Pangea. Thus, $X\left( 0 \right)$ =− 540 Myr and $X\left( 1 \right)$ =− 260 Myr. These results define $A\left( 0 \right) = 540\;Myr\;and\;A\left( 1 \right) = 180\;Myr$, which allows us to define the constant of acceleration of 3; $3 = A\left( 0 \right)/A\left( 1 \right)$, and thus $A\left( n \right) = \left( {\frac{540}{{3^{n} }}} \right)$. Thus, 540 Myr represents the first acceleration $A\left( 0 \right)$ of the supercontinent cycle. It should be noted that the constant 3 was obtained from the values of A(0) and A(1) and subsequently tested to obtain the following accelerations (A(2), A(3),…). Finally, all the investigations allow us to define the mathematical equation for the supercontinent cyclicity:

7 $$X\left( n \right) = 2*X\left( {n - 1} \right) - X\left( {n - 2} \right) - \left( {\frac{540}{{3^{n} }}} \right) with\, n \in {\mathbb{Z}} \left\{ {\begin{array}{*{20}c} { + \infty } \\ 0 \\ \end{array} } \right.$$

It is noteworthy that Eq. (7) produces the solution $X\left( 0 \right) = - 540 Myr$, considered as the first prediction when $X\left( { - 2} \right){ } = - 2000$ and $X\left( { - 1} \right){ } = - 1000{ }$ are used as the initial two assemblies.

## Supplementary Information

Supplementary Information.
