# Supplementary material for: Mathematical modelling reveals potential acceleration of the supercontinent cycle
Source: Sci Rep. 2022 Oct 17;12:17391. doi: 10.1038/s41598-022-21662-x (PMC9576751; doi:10.1038/s41598-022-21662-x)
Supplement: Supplementary file 1 — Supplementary Information. [file 41598_2022_21662_MOESM1_ESM.pdf]

Supplementary material S1: Mathematical modelling reveals potential acceleration of the supercontinent cycle

In this investigation, it is considered that the supercontinent cycle is coupled between assembly of megacontinent before the assemblage of a supercontinent e.g., [1]. To test the megacontinent hypothesis, Gondwana and Eurasia are removed from the supercontinent assemblies because they are considered as megacontinent by some researchers (see hypothesis in Table S1), [1,2]. Thus, only Columbia, Rodinia, Pangea and the future Pangea Proxima are considered as supercontinent and each assembly date is recovered from mathematical equation (1) results. This hypothesis allows us to obtain a logarithmic regression (8), (Figure S1). *X(n)* is still the supercontinent assembly and *n* is the position of the supercontinent in the sequence:

$$X(n) = 1573.5 * \ln(n) - 2025.2 \qquad R^2 = 0.997 \qquad (8)$$

First, this regression has a good correlation factor of R^2=0.997. It predicts Columbia, Rodinia, Pangea and Pangea Proxima at -2025, -935, -297 and 156 Myr (Table S1). These results are quite good fits with the initial hypothesis of acceleration of the supercontinent (mathematical equation (1)). In this equation supercontinent are recovered by natural integers (*n*=1;2;3;4;5) whereas megacontinent are represented by *n*=0.5;1.5;2.5;3.5;4.5 mimicking the assembly of a megacontinent before the complete assembly of a supercontinent. Therefore, it is recovered that megacontinent could form at -1387, -583, -54 and +341 Myr preceding Rodinia, Pangea and Pangea Proxima.

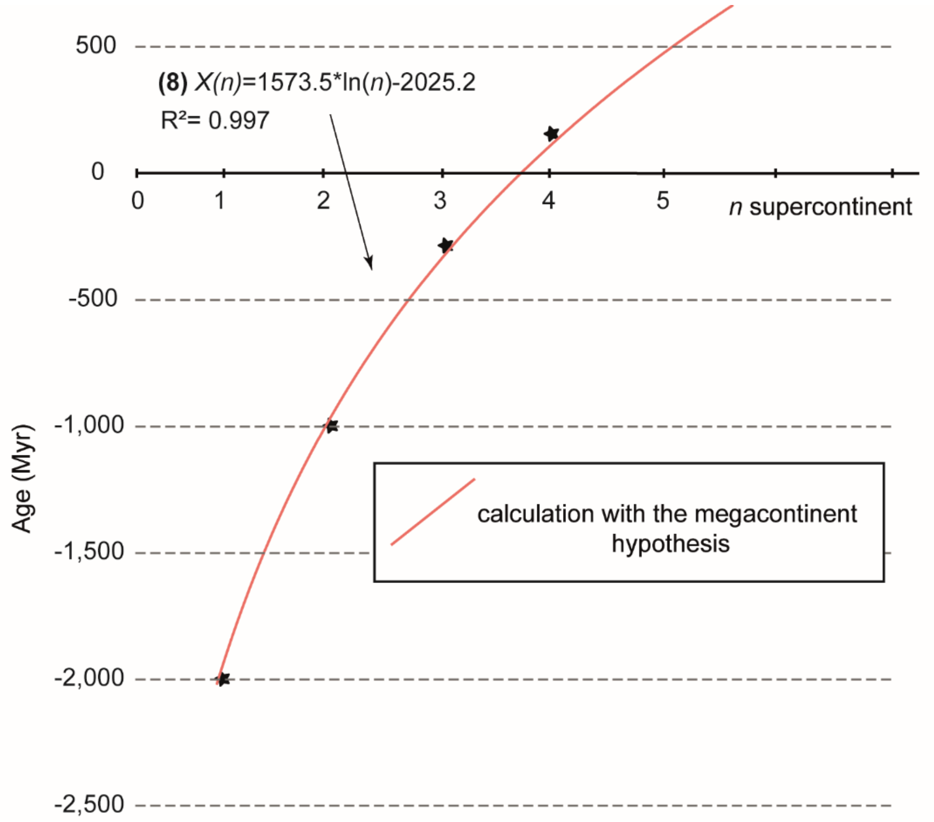

Figure S1 : Plot of age of assembly versus n supercontinent cycles. It shows that the mathematical equation (1) results for n= (1;2;3;4) are consistent with a logarithmic regression with a good correlation factor. The assembly results of the regression are portrayed in Table S1.

Table S1: Investigation on acceleration of the supercontinent cycle considering coupling between megacontinent and supercontinent (in Myr).

n supercontinent	assembly hypothesis	Equation (8): 1573.5*ln(*n*)-2025.2	supercontinent and mega continent	Equation (8): 1573.5*ln(*n*)-2025.2	*n* supercontinent cycle (Sc)	supercontinent cycle duration (Sc)
1	-2000	-2025	1	-2025		
2	-1000	-935	1.5	-1387		
3	-260	-297	2	-935	Sc(1)	1091
4	160	156	2.5	-583		
			3	-297	Sc(2)	638
			3.5	-54		
			4	156	Sc(3)	453
			4.5	341		
			5	507	Sc(4)	351

Interestingly, these hypotheses produce an assembly of megacontinent for Gondwana (-583 Myr) and Eurasia (-54 Myr) preceding Pangea and probably Pangea Proxima. These results could match the observation in Earth's history [1]. These assemblies are close to the supercontinent assemblies obtained by mathematical equation (1) at -540 and -40 Myr. However, the important findings about this investigation are not that Gondwana and Eurasia could be considered as megacontinent but that the investigation still reveals a probable acceleration of the supercontinent cycle (see Table S1). Indeed, the calculated supercontinent cycles (Sc) are Sc(1)=1091; Sc(2)=638; Sc(3)=453 and Sc(4)=351. Such supercontinent cycles clearly show an acceleration for supercontinent assembly although the acceleration is not constant in comparison with the findings of mathematical equation (1). More importantly, this investigation predicts an assembly of Pangea Proxima at +156 Myr consistent with the results of mathematical equation (1).

Therefore, it is concluded that whether considering supercontinent or megacontinent in the calculation of the supercontinent cyclicity, it still reveals a probable acceleration of the supercontinent cycle and an assembly of Pangea Proxima at approximately +160 Myr. These findings are in good agreement with the initial results of the mathematical equation (1).

References:

1 Wang, C., Mitchell, R. N., Murphy, J. B., Peng, P. & Spencer, C. J. The role of megacontinents in the supercontinent cycle. *Geology*, doi:http://10.1130/G47988.1 (2020).

2 Liu, Y., Mitchell, R. N., Brown, M., Johnson, T. E. & Pisarevsky, S. Linking metamorphism and plate boundaries over the past 2 billion years. *Geology*, doi:http://10.1130/g49637.1 (2022).
